# Metabolic Flux Analysis of *Xanthomonas oryzae* Treated with Bismerthiazol Revealed Glutathione Oxidoreductase in Glutathione Metabolism Serves as an Effective Target

**DOI:** 10.3390/ijms252212236

**Published:** 2024-11-14

**Authors:** Hai-Long Yu, Xiao-Long Liang, Zhen-Yang Ge, Zhi Zhang, Yao Ruan, Hao Tang, Qing-Ye Zhang

**Affiliations:** Hubei Key Laboratory of Agricultural Bioinformatics, College of Informatics, Huazhong Agricultural University, Wuhan 430070, China

**Keywords:** genome-scale metabolic network, inhibitor, target discovery, virtual screening

## Abstract

Bacterial blight (BB) of rice caused by *Xanthomonas oryzae* pathovar oryzae (*Xoo*) is a serious global rice disease. Due to increasing bactericide resistance, developing new inhibitors is urgent. Drug repositioning offers a potential strategy to address this issue. In this study, we integrated transcriptional data into a genome-scale metabolic model (GSMM) to screen novel anti-*Xoo* targets. Two RNA-seq datasets (before and after bismerthiazol treatment) were used to constrain the GSMM and simulate metabolic processes. Metabolic fluxes were calculated using parsimonious flux balance analysis (pFBA) identifying reactions with significant changes for target screening. Glutathione oxidoreductase (GSR) was selected as a potential anti-*Xoo* target and validated through antibacterial experiments. Virtual screening based on the target identified DB12411 as a lead compound with the potential for new antibacterial agents. This approach demonstrates that integrating metabolic networks and transcriptional data can aid in both understanding antibacterial mechanisms and discovering novel drug targets.

## 1. Introduction

Rice is one of the most important food sources for more than half of the world’s population, especially in Asia. With the development and application of the green revolution technology of modern crop breeding, rice yield has increased dramatically over the past four decades. In spite of great advances in food grain production, a considerable number of people still suffer from inadequate food due to climate change, population explosion, and rice disease. By conservative estimation, an approximate 20% increase in rice production is required to meet the needs of the predicted population by 2030. Reducing rice diseases, especially rice bacterial blight (BB), is one of the most effective methods to increase rice yield [1,2].

BB is one of the most serious diseases of rice caused by *Xanthomonas oryzae* pathovar oryzae *(Xoo).* In addition to reducing rice annual yield, BB disease also negatively affects grain quality by disrupting maturity [3,4] *Xoo*, as a Gram-negative bacterium, enters the xylem through leaf or root wounds and moves to leaves through the veins. The infected leaves of rice become faded and necrotic gradually from the leaf tip down along the veins and margins [5,6,7]. The disease-resistant breed cultivation and chemical controls (such as new bactericides) are used to combat *Xoo*. Bismerthiazol is a widely and commonly used inhibitor against *Xoo* [8,9]. Bismerthiazol not only induces plants to acquire resistance but also kills bacteria directly in vitro and in vivo. However, with the widespread use of this drug, the resistance of *Xoo* to bismerthiazol is becoming increasingly serious. The sensitivity of *Xoo* has significantly decreased in different regions, and resistant populations are continually increasing. This situation significantly impacts the effectiveness of bismerthiazol and poses greater challenges for rice cultivation [10,11]. Moreover, studies have indicated that bismerthiazol mitigates the pathogenicity of *Xoo* by targeting the histidine degradation pathway (Hut pathway) and quorum sensing mechanisms [12]. Due to its potential multi-target action, a comprehensive investigation into its specific molecular mechanisms and prospective targets could yield novel insights and effective strategies for drug development to combat the *Xoo* challenge.

The metabolic processes allow organisms to grow and reproduce, maintain structure, and respond to environmental changes. The metabolic process in cells can be reflected by a genome-scale metabolic model (GSMM) [13,14] in system biology, and GSMM can display the interaction between compounds and enzymes. GSMM is a comprehensive representation of the metabolic network, encompassing most of the enzyme-catalyzed biochemical reactions occurring in cells, which are interconnected through the metabolites involved [15]. In GSMM, each reaction is catalyzed by one or more enzymes coded by specific genes; thus, GSMM can be established through direct gene–protein-reaction connectivity. By GSMM network analysis, (1) the phenotype change could be predicted based on the relationship between phenotype and genotype in the network under different external conditions; (2) the optimal pathways could be determined to obtain more metabolites needed by metabolic engineering; (3) the flux distribution of intracellular biochemical reactions could be predicted [16]. To manually construct and curate hundreds of GSMM is extremely time-consuming. With the development of high-throughput sequencing and genome-scale metabolic model-reconstruction tools, it is possible to quickly and automatically construct GSMM for various species. Since the first GSMM for Escherichia coli was published in 1993, over 6000 models have been developed for a diverse array of organisms, encompassing prokaryotes, eukaryotes, and archaea [17]. These models have been extensively utilized in the study of organismal metabolic characteristics. Metabolic network-reconstruction tools such as CarveMe, ModelSEED, RAVEN, and Kbase are widely used for constructing GSMMs. Among them, CarveMe is most similar to manually constructed models [18,19,20,21,22].

Building on the foundation provided by GSMM, the simulation of metabolic fluxes becomes a crucial next step to understand and optimize metabolic processes; flux balance analysis (FBA) can calculate the optimal solution of an objective function under constrained conditions; thus, it has been widely used to analyze metabolic networks [13]. However, the inherent limitation of FBA is that it may yield multiple optimal solutions due to the presence of redundant pathways capable of satisfying the same objective. To delineate the range of possible flux states for each metabolic reaction, Flux Variability Analysis (FVA) is used to establish the boundaries of flux, thereby elucidating the robustness of the metabolic network and its capacity for alternative pathways. Additionally, stochastic metabolic network sampling generates a comprehensive set of feasible flux distributions, providing insights into the network’s potential adaptability and variability under diverse physiological conditions. To refine the solutions provided by FBA and address its redundancy, parsimonious flux balance analysis (pFBA) integrates an efficiency criterion aimed at minimizing the total metabolic flux across the network while still achieving the desired objective functions, such as maximizing growth rate [23,24,25]. The pFBA has been widely applied in the simulation of metabolic fluxes, such as in the work of Abhijit Paul et al. who used pFBA to identify potential cancer drug targets, and Zoran Nikoloski et al. who simulated metabolic changes in Arabidopsis under different environmental conditions [26,27]. Due to its ability to provide unique solutions and reproducibility, pFBA has become an important tool for studying metabolic networks. With the development of next-generation sequencing technology, gene-expression data have become easily available. The gene-expression process is closely related to metabolic processes [28,29]. Transcriptional integration algorithms, such as the integrative metabolic analysis tool (iMAT), E-Flux2, and Gene Inactivity Moderated by Metabolism and Expression (GIMME), can incorporate transcriptomic data into GSMM, enhancing the predictive capacity of metabolic models [30,31,32,33].

To explore the anti-*Xoo* mechanism of bismerthiazol and identify new antibacterial targets, the GSMM of *Xoo* was reconstructed based on its annotated genome. The RNA-seq data of *Xoo* treated with or without bismerthiazol were, respectively, integrated into the GSMM to simulate the corresponding metabolic process. The metabolic flux of each reaction in *Xoo* was calculated using pFBA. The potential antibacterial agent targets were screened based on the reactions with significantly differential metabolic flux. The antibacterial experiment was performed to verify the effectiveness of the selected target. Subsequently, based on the selected target, virtual screening was performed to identify novel inhibitors against *Xoo*, and one compound was found to be a lead compound with potential to be developed into a new antibacterial drug.

## 2. Result and Discussion

### 2.1. GSMM Reconstruction and FBA Analysis

The annotated genome files of *Xoo* were downloaded from National Center for Biotechnology (NCBI) (https://www.ncbi.nlm.nih.gov/nuccore/AE013598.1, accessed on 31 January 2014), to construct the GSMM by CarveMe [18]. The obtained GSMM of *Xoo* (*Xoo*-GSMM) included 2155 reactions, 1501 metabolites, and 730 genes. Based on the reconstructed *Xoo*-GSMM model, the pFBA analysis was performed.

**Figure 1:**
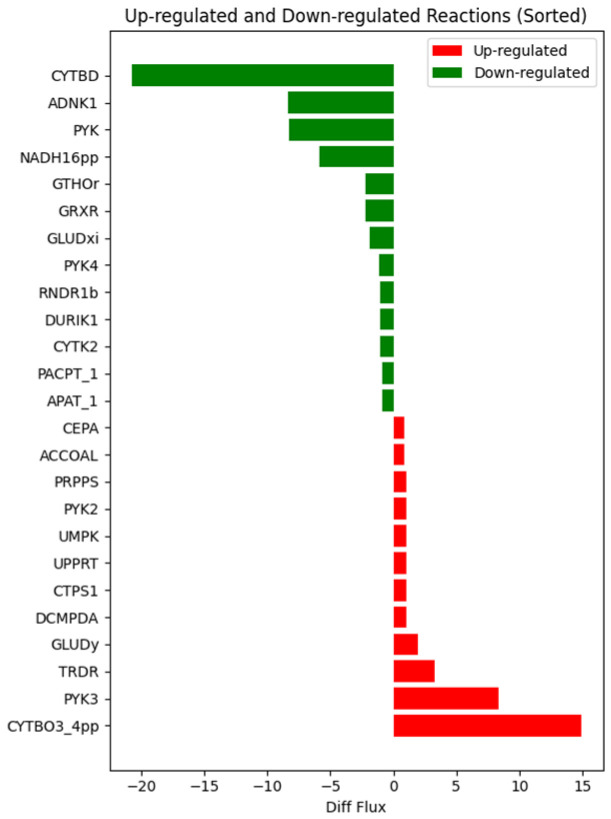
The 25 reactions with the largest differential metabolic flux change after *Xoo* was treated with bismerthiazol. Twelve reactions where differential metabolic flux was increased (red column) and 13 reactions where differential metabolic flux was reduced (green column).

To explore the potential mechanism of action of bismerthiazol against *Xoo*, RNA-seq data of *Xoo* treated with or without bismerthiazol were integrated into *Xoo*-GSSM and analyzed using the iMAT algorithm. The pFBA method was used to calculate the reaction flux and the flux differences between the two groups. Out of the 2155 reactions in the *Xoo*-GSMM, 1466 reactions corresponded to genes in the RNA-seq data. Among these 1466 reactions, the metabolic flux of 286 reactions changed in *Xoo* treated with bismerthiazol. Of these, 97 reactions exhibited increased metabolic flux, while 189 reactions displayed decreased flux. After removing transport reactions, non-target reactions, and loop reactions, the top 25 reactions with the largest changes in metabolic flux (including 12 upregulated and 13 downregulated reactions) were selected for further analysis. The names and differential metabolic flux data of these 25 reactions are shown in Figure 1. Their metabolic flux, corresponding genes, functions, and metabolic pathways are summarized in Tables S1 and S2.

**Figure 2:**
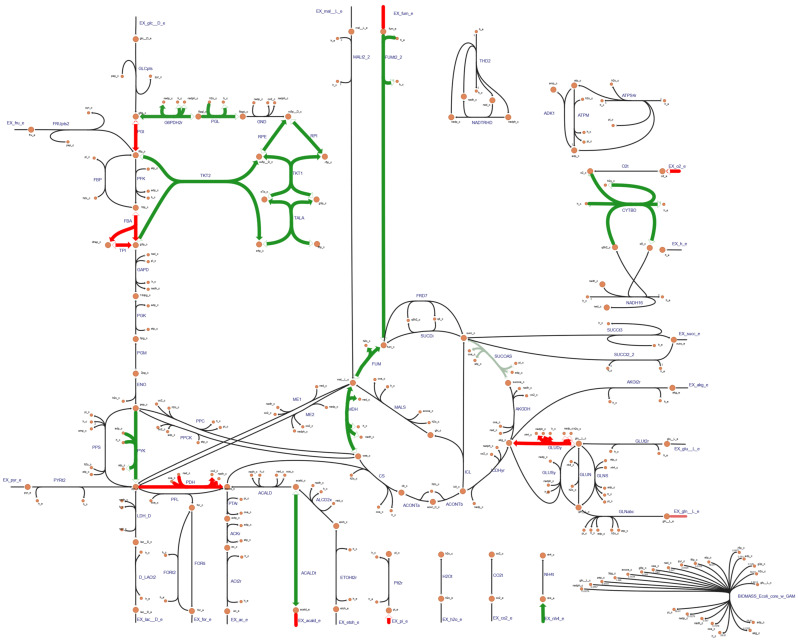
Visualization for the metabolic flux change of the core metabolism of *Xoo*-GSMM by Escher.

To display the metabolic flux change in each reaction intuitively, a web-based tool Escher was used to visualize the core data of reactions in the *Xoo*-GSMM. The reactions with the increased metabolic flux are indicated with red lines and those with the decreased metabolic flux are denoted with green lines (Figure 2).

### 2.2. Mechanism Analysis and Potential Target Discovery

Previous studies have proposed that the mechanism of action of bismerthiazol involves the hut pathway, motility regulation, stress responses, and biofilm synthesis [12]. Our results indicate that many reactions with significantly changed metabolic flux, such as CYTBO3_4pp, CYTBD, and NADH16pp, are related to motility regulation, stress responses, and biofilm synthesis. These findings are consistent with previously reported studies. Additionally, our results suggest that these reactions are also associated with carbon metabolism, glutathione metabolism, amino sugar and nucleotide sugar metabolism, deamination metabolism, purine metabolism, 2-oxocarboxylic acid metabolism, and electron transport and energy generation. These newly discovered pathways may be related to the anti-*Xoo* mechanism of bismerthiazol, warranting further investigation.

Among the top 25 reactions with the largest metabolic flux changes, the catalytic enzymes of 8 reactions have already been used as drug targets. Of these 8 reactions (PYK, PYK2, PYK3, PYK4, UPRRT, PRPPS, RNDR1b, and GTHOr), the catalytic enzymes of 7 reactions (PYK, PYK2, PYK3, PYK4, UPRRT, PRPPS, and RNDR1b) have been reported as antibacterial targets. The catalytic enzymes corresponding to PYK, PYK2, PYK3, and PYK4-pyruvate kinase and its variants have already been recognized as novel targets for controlling *Xoo* [34], validating the reliability of our integrative method that incorporates RNA sequencing data into GSMM. As the catalytic enzyme of the PRPPS reaction, phosphoribosylpyrophosphate synthetase (PRPP synthetase) catalyzes the reaction between ribose-5-phosphate and adenosine triphosphate (ATP) to produce phosphoribosyl pyrophosphate (PRPP) and pyrophosphate (PPi). This enzyme plays a pivotal role in regulating nucleotide synthesis, making it a crucial antibiotic target [35]. For the UPRRT reaction, uracil phosphoribosyltransferase catalyzes the conversion of uracil and PRPP into uridine monophosphate (UMP) and PPi, a key step in the pyrimidine nucleotide synthesis pathway. This enzyme has already been identified as a potent antibacterial target [36]. Additionally, ribonucleoside-diphosphate reductase, the enzyme catalyzing the RNDR1b reaction, is responsible for converting ribonucleotides to deoxyribonucleotides, essential components for DNA replication and repair. As a result, this enzyme is viewed as an ideal target for anti-proliferative compounds, applicable to eukaryotic cells (such as cancer cells), parasites, viruses, and bacteria, by inhibiting cellular replication [37].

The reaction formula of reaction GTHOr is as follows:

GTHOr:2 glutathione + NADP (+) <=> glutathione disulfide + NADPH + H^+^

The catalytic enzymes of GTHOr were glutathione oxidoreductase (GSR) [EC 1.8.1.7]. GSR is involved in mediating drug resistance associated with the redox homeostasis, and it is a potential therapeutic target for glioblastoma multiforme [38]. However, no report on the clear association of GSR with antibacterial target is available. Parthenolide (PTL) is a sesquiterpene lactone with multiple bioactivities and can completely inhibit the growth of *Xoo* with reducing GSR activity, suggesting that GSR is likely to be a potential drug target against *Xoo* [26].

Among the 25 reactions exhibiting significant changes, excluding the 8 reactions previously discussed, several other reactions occupy critical roles within the core metabolic pathways of bacteria, thereby offering substantial clinical relevance and commercial potential. For instance, reactions such as CYTBO3_4pp and CYTBD, which involve cytochrome oxidase, are pivotal in maintaining bacterial respiratory chain functionality, while GRXR and TRDR participate in the glutathione metabolism pathway, a key mechanism for bacterial oxidative stress management. The pronounced changes observed in these reactions underscore their potential as valuable targets in the development of novel antibacterial therapeutics.

### 2.3. Effectiveness Test of Target GSR

To verify whether the GSR from *Xoo* as an antibacterial target against *Xoo*, this experiment utilized reported GSR inhibitors for *Xoo* bactericidal tests. Although some GSR inhibitors might have lower toxicity or stronger inhibition effects, they were excluded due to multi-target effects or unclear mechanisms. For example, ethacrynic acid, fluoxetine, and auranofin might affect other biological pathways, creating non-specific effects, and some mechanisms targeting GSR are uncertain. Therefore, menadione and carmustine were chosen due to their clearer mechanisms: menadione binds GSR’s active site, disrupting cysteine residues to inhibit reduction activity, while carmustine alkylates GSR for irreversible inactivation, further weakening bacterial antioxidant capacity. PTL was selected as a positive control to validate experimental results [39,40,41,42,43].

**Figure 3:**
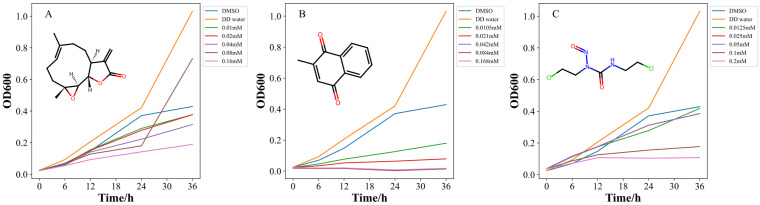
The turbidimetric growth curve of *Xoo* at different concentrations of PTL (**A**), menadione (**B**), and carmustine (**C**).

The growth-inhibition curves of the three inhibitors are shown in Figure 3. The inhibitory effects of menadione on *Xoo* at concentrations of 0.0105 mM, 0.021 mM, 0.042 mM, 0.084 mM, and 0.168 mM, respectively, were examined. With the rising concentration of menadione, the bacterium inhibition rate was increased. The menadione at the concentration of 0.042 mM showed the highest inhibition rate (almost reaching 100%) against *Xoo* (Figure 3B). Under the same condition, inhibitory effects of carmustine on *Xoo* at 0.0125 mM, 0.0250 mM, 0.050 mM, 0.100 mM, and 0.200 mM, respectively, were investigated. The growth-inhibition curves showed that the bacterium-inhibition rate was increased with the rising concentration of the carmustine (Figure 3C). The inhibition rate of carmustine against *Xoo* was about 75% at the concentration of 0.20 mM. The inhibition curve trend of carmustine was very similar to that of PTL (Figure 3A). Menadione exhibited the strongest inhibitory activity on the growth of *Xoo* and its inhibitory effect was observed the earliest. The experimental results demonstrated that *Xoo*-GSR could be used as an anti-*Xoo* target, and that the two corresponding drugs (menadione and carmustine), which have strong inhibitory effects on the growth of *Xoo*, could be used as lead compounds. In order to identify more novel lead compounds for antibacterial agents, virtual screening was performed based on the structure of *Xoo*-GSR.

### 2.4. Inhibitor Screening Based on Xoo-GSR

#### 2.4.1. Homology Modeling of *Xoo*-GSR

**Figure 4:**
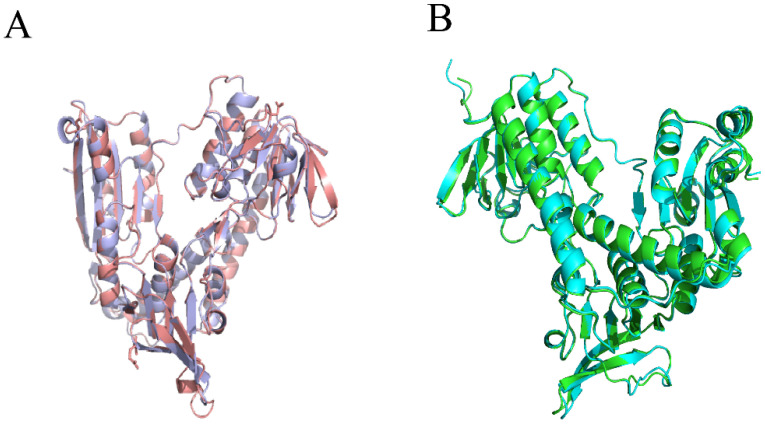
(**A**) Superposition of the homology model: *Xoo*-GSR (pink) with 1GER (purple). (**B**) Alignment of *Xoo*-GSR obtained from SWISS-MODEL (blue) and AlphaFold (green).

The detailed structure information of the target enzyme is critical for designing and screening potential inhibitors. Previous studies have documented that a reliable homology modeling could be performed when the sequence identity between two proteins exceeds 30% [44]. The sequence identity between our target (WP_101838026.1) and the template enzyme (Protein Data Bank (PDB) code: 1GER) was 45.77%, which guaranteed the quality of homology modeling. The three-dimensional structure of *Xoo*-GSR was generated by the web-based homology modeling server SWISS_MODEL [45,46], using the crystallographic structure of glutathione reductase (PDB code: 1GER) as the template. Figure 4A shows the superposition of the *Xoo*-GSR model with its template. As expected, the overall conformation of the model was very similar to that of the template. The stereochemical quality of this model was examined by PROCHECK. The results showed that the majority of the residues of the model occupied the most favorable regions in Ramachandran plots, and the information is summarized in detail in Table 1.

AlphaFold [47], developed by DeepMind, has significantly advanced the field of protein structure prediction through deep learning techniques. In this study, we utilized AlphaFold to predict the structure of the target protein and compared it with the previously predicted structure obtained from SWISS-MODEL. Figure 4B shows the comparison between the two structures. As expected, the structure predicted by AlphaFold aligned well with the model generated by SWISS-MODEL, demonstrating a high degree of structural overlap, which further confirms the reliability of the model. These results indicated that the obtained *Xoo*-GSR model was sufficiently reliable to be used for subsequent molecular docking analysis.

#### 2.4.2. Docking Analysis

**Figure 5:**
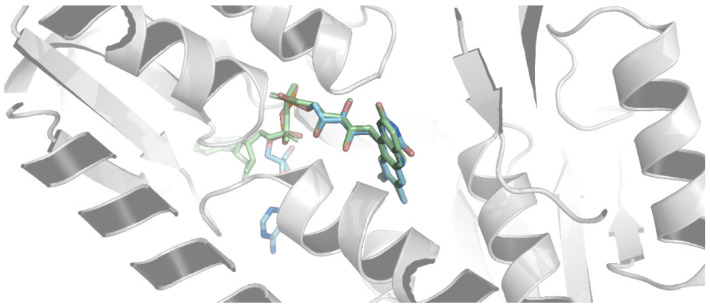
Schematic for the validation of molecular docking by “redocking” experiment: Docking conformation (green stick) versus original binding conformation for the ligand FAD (blue stick) in the active site of *Xoo*-GSR.

Molecular docking was performed using AutoDock Vina [48] to screen more lead compounds for the *Xoo*-GSR target. The *Xoo*-GSR model was pretreated with MGLTools by adding hydrogen, assigning atom types, and calculating charges. A redocking experiment was performed with the ligand flavin adenine dinucleotide (FAD) from 1GER as a test molecule to ensure that the docking algorithm could produce the observable binding mode. The initial geometric parameters of FAD were extracted from the 1GER crystal structure. FAD was added with hydrogen atoms and energy-minimized using the Tripos force field [49]. As shown in Figure 5, the binding mode of FAD to the *Xoo*-GSR target obtained by AutoDock Vina was almost identical to that of the 1GER crystal complex, indicating that our molecular docking was feasible for subsequent *Xoo*-GSR structure-based virtual screening.

The potential antibacterial agents were quickly obtained by virtual screening. The small molecule library was prepared by adding hydrogens, adding charges, assigning atom types, and setting up the “torsion tree” using the prepare_ligand4.py script from MGLTools. The parameters of the *Xoo*-GSR system were set as follows: center_x = 34, center_y = 16, center_z = 22, size_x = 16, size_y = 16, size_z = 16, num_modes = 10, and exhaustiveness = 8. FAD was added into the small molecule library as a control to calibrate the virtual screening process.

According to the docking score of FAD, compound binding mode at the active site, and structural diversity, the representative candidate compounds of *Xoo*-GSR (as shown in Table 2) were identified.

#### 2.4.3. Experimental Evaluation

Based on the compound structure characteristics and purchase availability, the compounds DB12411, DB15039, and DB11852 were selected to perform the anti-*Xoo* experiment. First, the pre-experiments with these three compounds against *Xoo* were performed at the concentration of 0.25 mM. The results showed that the inhibition rate of DB12411 against *Xoo* reached 97.08% and 99.23% at 6 h and 12 h post-treatment, respectively, indicating that DB12411 had good bactericidal activity against *Xoo*. The experimental results are summarized in Table 3.

Based on the pre-experiment results, the Minimum Inhibitory Concentration (MIC) value of DB12411 against *Xoo* was further examined at concentrations of 0.01 mM, 0.02 mM, 0.03 mM, 0.04 mM, and 0.05 mM. Dimethyl sulfoxide (DMSO) was used as a blank control in the experiment.

**Figure 6:**
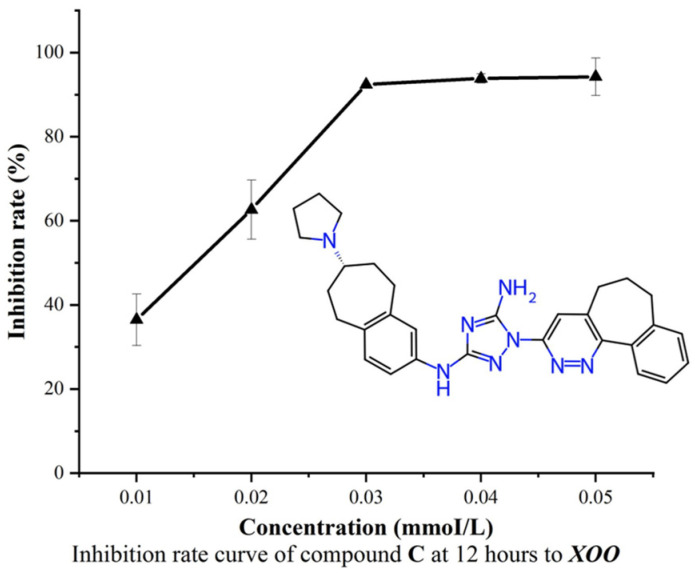
The inhibition rate curve of drug DB12411 against *Xoo* at different concentrations.

The bacterium-inhibition curves showed that with increasing compound concentration, the inhibitory rate increased (Figure 6). When the concentration reached 0.03 mM, the inhibitory rate of DB12411 against *Xoo* reached about 92.0% and became steady. The MIC of DB12411 against *Xoo* was 0.032 mM. As a comparison, the commercial agent bismerthiazol has been reported in multiple studies to have an MIC range of 0.14 mM to 0.65 mM against *Xoo* [50,51]. In contrast, DB12411 exhibits a significantly lower MIC than bismerthiazol, indicating stronger antibacterial potential. DB12411, also known as bemcentinib, has been used for treating non-small cell lung cancer, breast cancer, and COVID-19. To date, no report on its antibacterial effect is available. The above experimental results demonstrated that DB12411 could inhibit the growth of *Xoo*, thus making it a potential lead compound for anti-*Xoo* agents.

## 3. Materials and Methods

### 3.1. RNA-Seq Data Preprocessing

The raw sequence reads of RNA-seq data were downloaded from the Sequence Read Archive (SRA) database of the NCBI. The raw sequence reads of *Xoo* at 0 h and 4.5 h post treatment with 30 mg/L bismerthiazol were downloaded from the SRA database (with accession number of SRR3733119 and SRR3733118). The samples were sequenced on the Illumina Hiseq 2000 platform, and 150-bp paired-end reads were generated. The clean sequencing reads were obtained by removing low-quality reads. The paired-end clean reads were aligned to the reference genome (AE013598) using HISAT2 [52]. The transcript abundance of each gene was calculated using stringtie [53] software and converted into Fragments Per Kilobase of transcript per Million mapped reads (FPKM).

### 3.2. Automated Reconstruction and Analysis of GSMM

CarveMe [18] is a Python-based automated tool designed to reconstruct a metabolic network. This command-line tool requires only the annotated whole genome sequence of a species to begin its process. It utilizes a unique top-down approach, starting from annotated whole genome data and relying on a manually curated universal template model integrated with the Biochemical Genetic and Genomic knowledge base (BiGG) database of biochemical genetic and genomic data. This strategy enables CarveMe to efficiently identify and construct metabolic networks that include all known metabolic pathways and reactions, quickly generating models ready for various metabolic analyses. This method is particularly suitable for rapidly constructing precise metabolic models for specific species or multi-species microbial communities. The detailed reconstruction process of GSMM of *Xoo* referred to the previously reported method [45,46].

The iMAT uses trinary expression data as input. The trinary gene expression data (1/0/1) are mapped to metabolic reactions, determining the expression state of each reaction through Boolean mapping and generating subsets of high- and low-expression reactions. Then, mixed integer linear programming (MILP) is employed to find a steady-state flux distribution that satisfies stoichiometric and thermodynamic constraints while maximizing the consistency of reaction activity with their expression states. Finally, in the phase of determining reaction activity states, the MILP solver provides the optimal solution and calculates the similarity score with the expression data. Subsequently, this score is imposed as a constraint to explore the optimal solution space, and Flux Variability Analysis (FVA) is used to determine the minimum and maximum attainable flux for each reaction, thereby assessing the activity states of the reactions and elucidating the active pathways under the studied conditions.

The RNA-seq expression values were ranked, and the iMAT algorithm was employed to construct a context-specific metabolic network. The parameters for iMAT were defined as follows: the value at the 5th percentile was set as the lower threshold, and the value at the 65th percentile was set as the upper threshold. Reactions classified as low-expression reactions were those below the lower threshold, whereas reactions above the upper threshold were designated as high-expression reactions. In this process, the iMAT algorithm aims to retain high-expression reactions wherever possible, ensuring that these reactions remain active in the model and aligning the metabolic network with the gene expression data.

### 3.3. Antibacterial Experiment

To verify whether *Xoo*-GSR could be used as a target for a new antibiotic against *Xoo*, antibacterial experiments were performed. Menadione and carmustine (known inhibitors of GSR) were purchased from Aladdin, Shanghai, China. A whole-cell assay was conducted to evaluate the growth inhibition of *Xoo* using the broth microdilution procedure [54]. The compounds were dissolved in a dimethyl sulfoxide (DMSO) solution, and the DMSO concentration was maintained at 0.2% in all assays to avoid affecting *Xoo* growth. Various concentrations of compounds were added along with an equal volume of DMSO solvent as an untreated control. The concentration for 50% of maximal effect (EC_50_) and MIC values were determined under five different compound concentrations. The experiments at each concentration were conducted in triplicates. Bacterial survival rate was measured with a microtiter enzyme-linked immunosorbent assay (ELISA) reader at ELISA. Candidate compounds obtained by virtual screening were also purchased from Aladdin, Shanghai, China, and subjected to the same antibacterial experiments.

### 3.4. Virtual Screening of Xoo-GSR Targets

An accurate three-dimensional structure of the target is important for virtual screening of novel inhibitors. No crystal structures of GSR from *Xoo* were obtained by experiments, but several crystal structures of GSR from other bacteria were obtained from the Protein Data Bank. The target sequences of *Xoo*-GSR were submitted to the SWISS-MODEL [45,46] server for structural modeling. The stereochemical quality of homology modeling was examined by PROCHECK.

To identify novel candidate inhibitors of *Xoo*-GSR, molecular docking virtual screening was performed using AutoDock Vina. The homology model of *Xoo*-GSR was used as the receptor and prepared by adding hydrogen, assigning atom types, and calculating charges using MGLTools.

The small molecular library was obtained from the ZINC15 database. To improve the selection effectiveness of small molecules, the “World” set of drugs was used, which included drugs approved by at least one drug administration around the world. The prepare_ligand4.py script in AutoDock Tools was used to process the small molecules. Based on this script, the charge distribution of each molecule was calculated, and the root of torsion and rotatable bonds of each molecule were determined. The processed molecules were saved as PDBQT files.

## 4. Conclusions

BB caused by *Xoo* is one of the most serious rice diseases worldwide. In this study, to explore the possible anti-*Xoo* mechanism of bismerthiazol and identify the new antibacterial target against *Xoo*, the transcriptomic data from before and after bismerthiazol treatment were integrated into the GSMM to reconstruct a context-specific GSMM of *Xoo* and simulate the metabolic processes, identifying reactions with significant flux differences. The mechanisms related to the reactions with the large metabolic flux change were in agreement with previous reports, which confirmed the effectiveness of our integration method. Combining literature reports, the study identified the potential target *Xoo*-GSR, which exhibited metabolic flux change, and its effectiveness was further verified through antibacterial experiments. Virtual screenings were performed based on the *Xoo*-GSR target through molecular docking, and compound DB12411 (with a MIC of 0.032 mM against *Xoo*) was screened and verified as an anti-*Xoo* lead compound. Our results will provide a reference for revealing the antibacterial mechanism of other drugs and identifying more novel drug targets.

Genomic and transcriptomic data are widely used to identify new targets in pathogenic bacteria. However, relying solely on genomic and transcriptomic data has limitations in characterizing *Xoo*’s metabolic and pharmacological mechanisms, as these data are further removed from actual metabolic activities and may miss critical regulatory details. Although metabolomics methods can directly reflect changes at the metabolite level, the technology remains immature, costly, and limited in precision [55]. The integration of transcriptomic data with a GSMM used in this study is a widely applied and validated analytical approach, serving as an effective alternative to metabolomics to some extent. Integrating metabolic networks with transcriptomic data not only compensates for the limitations of transcriptomics but also provides a feasible and effective alternative for studying metabolic changes [56]. Additionally, to more accurately detect the metabolic changes in *Xoo* under bismerthiazol treatment, proteomics and metabolomics could also be employed; however, these methods typically require new experimental data, which involves considerable time and resources. Given the current data, combining transcriptomic data with GSMM would be the optimal approach to detect metabolic changes in *Xoo*.

Although this study primarily focused on GSR as the main research target, our analysis also revealed significant changes in the metabolic flux of several other reactions. Notably, the cytochrome oxidases CYTBD and CYTBO3_4pp exhibited the greatest changes in flux, indicating their crucial roles in the respiratory chain function and oxidative stress management of *Xoo*. The activity changes in these enzymes directly affect the bacterial energy metabolism and viability, making them potential targets against *Xoo*. Future research should explore whether CYTBD and CYTBO3_4pp could serve as new strategies against Xanthomonas oryzae pv. oryzae (*Xoo*) infection. This exploration should include systematic functional validation of these targets through gene knockout and overexpression experiments, as well as extensive in vivo tests to verify the efficacy and safety of potential drug candidates. By thoroughly understanding the roles of these targets in *Xoo* infections, we can lay a solid scientific foundation for developing new antibiotic strategies.

## Figures and Tables

**Table 1:** The stereochemical quality score of *Xoo*-GSR.

Model	Residues in Most Favoured Regions	Residues in Additional Allowed Regions	Residues in Generously Allowed Regions	Residues in Disallowed Regions
*Xoo*-GSR (378)	348 (92.1%)	24 (6.3%)	6 (1.6%)	0 (0.0%)

**Table 2:** The selected candidate compounds for *Xoo*-GSR by molecular docking analysis.

Molecular ID	Docking Score
GSR-DB12411	−12.3
FAD	−12.2
GSR-DB15039	−11.8
GSR-DB04888	−11.7
GSR-DB11852	−11.7
GSR-DB12886	−11.7

**Table 3:** The preliminary experimental evaluation results of the three compounds.

Molecular	CAS	Molecular Weight	Inhibition Rate (6 h)	Inhibition Rate (12 h)
DB12411	1037624-75-1	506.64	97.08%	99.23%
DB15039	1642303-38-5	605.56	45.20%	27.33%
DB11852	1000787-75-6	517.4	29.51%	13.74%
	DMSO		9.72%	6.39%

## Data Availability

The original contributions presented in the study are included in the article/Supplementary Materials, further inquiries can be directed to the corresponding author.

## Supplementary Materials

The following supporting information can be downloaded at: https://www.mdpi.com/article/10.3390/ijms252212236/s1.

## Abbreviations

BB, Bacterial blight; *Xoo*, *Xanthomonas oryzae* pathovar oryzae; GSSM, genome-scale metabolic model; FBA, flux balance analysis; pFBA, parsimonious flux balance analysis; MILP, mixed integer linear programming; iMAT, integrative metabolic analysis tool; GIMME, Gene Inactivity Moderated by Metabolism and Expression; GSR, Glutathione oxidoreductase; FAD, flavin adenine dinucleotide; PDB, Protein Data Bank; FVA, Flux Variability Analysis; MIC, minimum inhibitory concentration; DMSO, Dimethyl sulfoxide; SRA, Sequence Read Archive; EC_50,_ concentration for 50% of maximal effect; RNA sequencing, RNA-seq; BIGG, Biochemical Genetic and Genomic knowledgebase; NCBI, National Center for Biotechnology Information; UMP, uridine monophosphate; PRPP, phosphoribosylpyrophosphate synthetase; PPi, pyrophosphate; ATP, Adenosine triphosphate; FPKM, Fragments Per Kilobase of transcript per Million mapped reads; ELISA, Enzyme-linked immunosorbent assay.
