# Supplementary material for: Metabolic Flux Analysis of Xanthomonas oryzae Treated with Bismerthiazol Revealed Glutathione Oxidoreductase in Glutathione Metabolism Serves as an Effective Target
Source: Int J Mol Sci. 2024 Nov 14;25(22):12236. doi: 10.3390/ijms252212236 (PMC11594844; doi:10.3390/ijms252212236)
Supplement: Supplementary file 1 [file ijms-25-12236-s001.zip › ijms-3282234-supplementary.pdf]

Supporting Information

Metabolic Flux Analysis of *Xanthomonas oryzae* Treated with Bismerthiazol Revealed Glutathione Oxidoreductase in Glutathione Metabolism Serves as an Effective Target

Hai-Long Yu †, Xiao-Long Liang †, Zhen-Yang Ge, Zhi Zhang, Yao Ruan, Hao Tang and Qing-Ye Zhang *

Hubei Key Laboratory of Agricultural Bioinformatics, College of Informatics, Huazhong Agricultural University, Wuhan 430070, China;

* Correspondence: zqy@mail.hzau.edu.cn; Tel.: +86-27-8728-0877

† These authors contributed equally to this work.

Table S1. The information summary of the reactions with metabolic flux upregulation.

ID	Flux	Target name	Formula	Pathway
CYTBO3_4pp	14.884833	Cytochrome oxidase bo3 (ubiquinol-8: 4 protons) (periplasm)	4.0 h_c + 0.5 o2_c + q8h2_c ⇌ h2o_c + q8_c + 4.0 h_p	
PYK3	8.321336	Pyruvate kinase(3)	gdp_c + h_c + pep_c ⇌ gtp_c + pyr_c	Glycolysis / Gluconeogenesis<br>Pyruvate metabolism<br>Metabolic pathways<br>Biosynthesis of secondary metabolites<br>Microbial metabolism in diverse environments
TRDR	3.241137	Thioredoxin reductase (NADPH)	h_c + nadph_c + trdox_c ⇌ nadp_c + trdrd_c	Selenocompound metabolism
GLUDy	1.907948	Glutamate dehydro-genase (NADP)	glu__L_c + h2o_c + nadp_c ⇌ akg_c + h_c + nadph_c + nh4_c	Arginine biosynthesis<br>Alanine, aspartate and glutamate metabolism<br>Nitrogen metabolism; Metabolic pathways<br>Microbial metabolism in diverse environments
DCMPDA	1.068792	DCMP deaminase	dcmp_c + h_c + h2o_c ⇌ dump_c + nh4_c	Metabolic pathways<br>Pyrimidine metabolism<br>Nucleotide metabolism
CTPS1	1.06577	CTP synthase NH3	atp_c + nh4_c + utp_c ⇌ adp_c + ctp_c + 2.0 h_c + pi_c	Pyruvate metabolism<br>Metabolic pathways<br>Nucleotide metabolism<br>Biosynthesis of cofactors
UPPRT	1.063056	Uracil phosphoribosy-ltransferase	prpp_c + ura_c ⇌ ppi_c + ump_c	Pyrimidine metabolism<br>Metabolic pathways<br>Nucleotide metabolism
UMPK	1.061174	UMP kinase	atp_c + ump_c ⇌ adp_c + udp_c	Pyrimidine metabolism<br>Metabolic pathways<br>Nucleotide metabolism<br>Biosynthesis of cofactors

PYK2	1.059291	Pyruvate kinase(3)	h_c + pep_c + udp_c ⇌ pyr_c + utp_c	Glycolysis / Gluconeogenesis<br>Pyruvate metabolism<br>Metabolic pathways<br>Biosynthesis of secondary metabolites<br>Microbial metabolism in diverse environments
PRPPS	1.057372	Phosphoribosylpyrophosphate synthetase	atp_c + r5p_c ⇌ amp_c + h_c + prpp_c	Pentose phosphate pathway<br>Purine metabolism<br>Metabolic pathway<br>Biosynthesis of secondary metabolites<br>Microbial metabolism in diverse environments<br>Carbon metabolism； Biosynthesis of amino acids
ACCOAL	0.901765	Acetate-CoA ligase (ADP-forming)	atp_c + coa_c + ppa_c ⇌ adp_c + pi_c + ppcoa_c	Glycolysis / Gluconeogenesis<br>Pyruvate metabolism<br>Metabolic pathways<br>Propanoate metabolism Microbial metabolism in diverse environments
CEPA	0.866570	Cellobiose phosphorylase	pi_c + cellb_c ⇌ g1p_c + glc__D_c	Starch and sucrose metabolism<br>Metabolic pathways

Table S2. The information summary of the reactions with metabolic flux downregulation.

ID	Flux	Target name	Formula	Pathway
CYTBDt	-20.748077	Cytochrome oxidase bd (ubiquinol-8: 2 protons)	2.0 h_c + 0.5 o2_c + q8h2_c ⇌ h2o_c + q8_c + 2.0 h_e	
ADNK1	-8.329232	Adenosine kinase	adn_c + atp_c ⇌ adp_c + amp_c + h_c	Purine metabolism<br>Metabolic pathways<br>Nucleotide metabolism
PYK	-8.283889	Pyruvate kinase	adp_c + h_c + pep_c ⇌ atp_c + pyr_c	Glycolysis / Gluconeogenesis<br>Pyruvate metabolism<br>Metabolic pathways<br>Biosynthesis of secondary metabolites<br>Microbial metabolism in diverse environments
NADH16pp	-5.856512	NADH dehydrogenase (ubiquinone-8 & 3 protons) (periplasm)	4.0 h_c + nadh_c + q8_c ⇌ nad_c + q8h2_c + 3.0 h_p	Arginine biosynthesis<br>Alanine, aspartate and glutamate metabolism<br>Nitrogen metabolism; Metabolic pathways<br>Microbial metabolism in diverse environments
GRXR	-2.173855	Glutaredoxin reductase	grxox_c + 2.0 gthrd_c ⇌ grxrd_c + gthox_c	Glutathione metabolis<br>Metabolic pathways
GTHOr	-2.173855	Glutathione oxidoreductase	gthox_c + h_c + nadph_c ⇌ 2.0 gthrd_c + nadp_c	Glutathione metabolis<br>Metabolic pathways
GLUDxi	-1.885036	Glutamate dehydrogenase NAD	glu__L_c + h2o_c + nad_c ⇌ akg_c + h_c + nadh_c + nh4_c	Pyrimidine metabolism
PYK4	-1.089009	UMP kinase	atp_c + ump_c ⇌ adp_c + udp_c	Pyrimidine metabolism<br>Metabolic pathways<br>Nucleotide metabolism<br>Biosynthesis of cofactors

PYK2	-1.059291	Pyruvate kinase(3)	h_c + pep_c + udp_c ⇌ pyr_c + utp_c	Glycolysis / Gluconeogenesis<br>Pyruvate metabolism<br>Metabolic pathways<br>Biosynthesis of secondary metabolites<br>Microbial metabolism in diverse environments
PRPPS	-1.057372	Phosphoribosylpyrophosphate synthetase	atp_c + r5p_c ⇌ amp_c + h_c + prpp_c	Pentose phosphate pathway<br>Purine metabolism<br>Metabolic pathway<br>Biosynthesis of secondary metabolites<br>Microbial metabolism in diverse environments<br>Carbon metabolism；Biosynthesis of amino acids
ACCOAL	-0.901765	Acetate-CoA ligase (ADP-forming)	atp_c + coa_c + ppa_c ⇌ adp_c + pi_c + ppcoa_c	Glycolysis / Gluconeogenesis<br>Pyruvate metabolism<br>Metabolic pathways<br>Propanoate metabolism Microbial metabolism in diverse environments
CEPA	-0.866570	Cellobiose phosphorylase	pi_c + cellb_c ⇌ g1p_c + glc__D_c	Starch and sucrose metabolism<br>Metabolic pathways
